# Ethanol-mediated activation of the NLRP3 inflammasome in iPS cells and iPS cells-derived neural progenitor cells

**DOI:** 10.1186/s13041-016-0221-7

**Published:** 2016-05-10

**Authors:** Lidia De Filippis, Apoorva Halikere, Heather McGowan, Jennifer C. Moore, Jay A. Tischfield, Ronald P. Hart, Zhiping P. Pang

**Affiliations:** Child Health Institute of New Jersey, Rutgers University-Robert Wood Johnson Medical School, room 3233D, 89 French Street, New Brunswick, NJ 08901 USA; Department of Neuroscience and Cell Biology, Rutgers University-Robert Wood Johnson Medical School, room 3233D, 89 French Street, New Brunswick, NJ 08901 USA; Department of Genetics, Rutgers University, Piscataway, 08854 USA; Human Genetic Institute of New Jersey, Rutgers University, Piscataway, 08854 USA; Department of Cell Biology and Neuroscience, Rutgers University, Piscataway, 08854 USA

**Keywords:** Stem cells, Alcohol use disorders, Neuroinflammation, Human induced pluripotent stem cells, Disease modeling

## Abstract

**Background:**

Alcohol abuse produces an enormous impact on health, society, and the economy. Currently, there are very limited therapies available, largely due to the poor understanding of mechanisms underlying alcohol use disorders (AUDs) in humans. Oxidative damage of mitochondria and cellular proteins aggravates the progression of neuroinflammation and neurological disorders initiated by alcohol abuse.

**Results:**

Here we show that ethanol exposure causes neuroinflammation in both human induced pluripotent stem (iPS) cells and human neural progenitor cells (NPCs). Ethanol exposure for 24 hours or 7 days does not affect the proliferation of iPS cells and NPCs, but primes an innate immune-like response by activating the NLR family pyrin domain containing 3 (NLRP3) inflammasome pathway. This leads to an increase of microtubule-associated protein 1A/1B-light chain 3^+^ (LC3B^+^) autophagic puncta and impairment of the mitochondrial and lysosomal distribution. In addition, a decrease of mature neurons derived from differentiating NPCs is evident in ethanol pre-exposed compared to control NPCs. Moreover, a second insult of a pro-inflammatory factor in addition to ethanol preexposure enhances innate cellular inflammation in human iPS cells.

**Conclusions:**

This study provides strong evidence that neuronal inflammation contributes to the pathophysiology of AUDs through the activation of the inflammasome pathway in human cellular models.

## Background

Alcohol use disorders (AUDs) are among the most common pathologies that affect the central nervous system (CNS). Fetal exposure to ethanol is known to cause long-term cognitive impairment and brain deficits [1, 2] that are commonly referred to as Fetal Alcohol Spectrum Disorders (FASDs). Despite a wide array of epidemiological studies that have investigated the genetic predisposition to develop AUDs, the cellular underpinnings and the pathophysiology of AUDs remain elusive in the CNS [3].

Ethanol is known to act as a powerful epigenetic disruptor and is potentially able to interfere with cellular metabolism and differentiation. In particular, neuroinflammation and oxidative damage of mitochondria and cellular proteins are thought to contribute to the progression of neurological disorders initiated by alcohol abuse [4]. Ethanol can initiate an innate immune-like response in the CNS [5] via two main receptors and their respective signaling pathways: the membrane bound toll-like receptors (TLRs) [6] and the cytoplasmic NOD-like receptor family, pyrin domain containing 3 (NLRP3). NLRP3 forms intracellular danger-sensing multi-protein platforms called inflammasomes [5]. Activation of both the TLR- and NLRP3-mediated pathways in mammals is correlated with aging [7, 8] and cellular insults, including ethanol exposure [5]. NLRP3 can activate inflammatory caspases, e.g. Caspase-1 (Casp1), which accelerates the aging process through the impairment of autophagy, thus eventually leading to cell death [9]. On the other hand, ethanol has also been shown to induce the activation of Caspase-3 (Casp3)-dependent apoptosis and necrosis in vivo [10]. However, whether ethanol exposure activates these cellular inflammatory pathways in human cells is not clear.

Given the inaccessibility of human neural tissue, human induced pluripotent stem (iPS) cell-derived neurons and neural progenitor cells (NPCs) represent powerful tools for testing the effects of ethanol on both early brain development and neuronal differentiation in vitro [11]. The inconclusive and controversial findings of previous studies exploring the effects of ethanol on NPCs could be attributed to varying culture methods, approaches, as well as model systems [3, 12, 13]. Furthermore, the intrinsic variability between iPS cell lines derived from different individuals can also contribute to the incongruities in these studies [14].

In order to model the pathogenesis of AUDs with limited intrinsic variability, we have focused our analysis on the effects of alcohol on cells derived from the same individual at three different stages: pluripotency (i.e. iPS cells), neurogenesis (i.e. NPCs), and terminal differentiation (i.e. post-mitotic neurons) [11]. In accordance with previous studies on postmortem human brains [15, 16], we show that neither acute (24 hours) nor prolonged (7 days) exposure to 70mM ethanol affects the proliferation or self-renewal of iPS cells or NPCs, but most likely impacts terminal differentiation and neuronal function. More importantly, we show an alteration of the mitochondrial pattern, as well as activation of the NLRP3 inflammasome pathway in these cells in response to ethanol exposure [5, 17]. This finding is consistent with the development of a remarkable neuroinflammatory environment in the brains of patients with a history of long-term alcohol dependence [4, 11, 18].

## Results

### Ethanol activates the inflammasome pathway in iPS cells

In order to model the effects of ethanol in humans, we used an iPS cellular model derived from individuals with no known alcohol dependence. We chose to treat the cells for either 24 hours (24hr, acute) or 7 days (7d, prolonged) with a dose of ethanol (70mM) that is consistent with the blood alcohol content following an episode of binge drinking or chronic alcohol consumption (Centers for Disease Control and Prevention (CDC), 2004) [19] and that triggers cortical neurodegeneration in fetal alcohol syndrome [20].

**Fig. 1:**
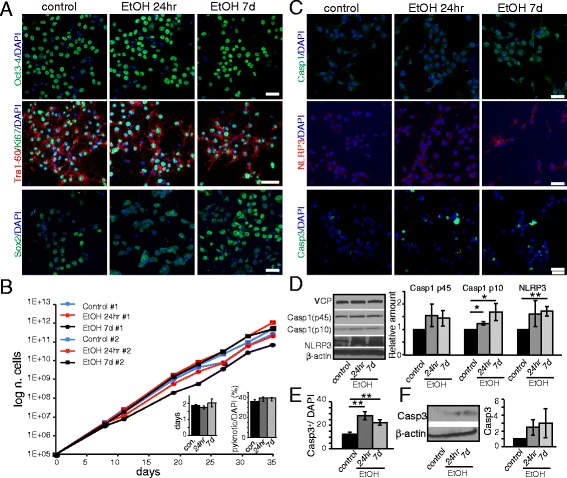
Ethanol activates the inflammasome pathway in iPS cells. iPS cells were cultured in 70 mM ethanol for 24hr or 7d. **a** From the top: Immunofluorescence analysis by confocal microscopy shows the expression of pluripotency markers Oct3-4, Tra-1-60, Sox2, and of the proliferation marker Ki-67 in iPS cells after 24hr or 7d ethanol exposure. Scale bar: 50 μm. **b** Growth curve of iPS cell lines #1 and #2. *Inserts*: showing the doubling time (days in vitro) (*left*), and the relative percentage of pyknotic nuclei over total DAPI^+^ nuclei (*right*). **c** Confocal microscopy images showing the expression of the apoptotic marker, Cleaved Caspase-3 (Casp3) and of the inflammasome-related markers, Caspase-1 (Casp1) and NLRP3. Scale bars: 50 μm. **d** Western Blot analysis and graph showing the relative densitometric analysis of expression of the inflammasome-pathway markers Casp1 (p45 and p10) and NLRP3. Quantification of the proteins was normalized to β-actin expression. **e** Graph showing the relative percentage of Casp3^+^ cells over the total number of DAPI^+^ cell nuclei. **f** Western Blot analysis of Casp3 expression

Ethanol exposure has reportedly altered the expression of pluripotency markers during early differentiation in murine embryonic stem cells [21]. As such, we first asked whether ethanol impacts the pluripotency of human iPS cells. We analyzed the expression of Oct4, Tra-1-60 and Sox2 by immunohistochemistry (IHC). Interestingly, no abnormal pattern was detectable in treated compared to untreated cells, suggesting that pluripotency likely remains unaffected (Fig. 1a).

To assess if ethanol affects the proliferation and survival of iPS cells, we performed a growth curve analysis and calculated their doubling time over passaging (Fig. 1b and 1b insert left, n = 4 lines). To our surprise, no significant difference was observed between ethanol - exposed and control cells (Fig. 1b). In addition, there was no change in the expression of the proliferation marker Ki67 (Fig. 1a) or in pyknotic nuclei (Fig. 1b insert right) between treated and untreated cells, suggesting that ethanol exposure (24hr or 7d) does not impact the survival or proliferation of iPS cells.

In vivo studies of the mouse cerebral cortex have suggested that ethanol induces neuronal damage through neuroinflammation, specifically through the activation of the inflammasome–mediated pathway [5, 6]. To assess whether ethanol activates the same pathway in human cells in vitro, we evaluated the expression of NLRP3 and Casp1 in ethanol-treated and untreated iPS cells (Fig. 1c–e). We showed by IHC and Western Blot analysis that the amounts of activated Casp1 and NLRP3 were increased in iPS cells after ethanol exposure (Fig. 1d). Conversely, the levels of pro-caspase- 1 (p45) were not significantly altered (Fig. 1d), suggesting that ethanol drives the activation of Casp1 with no substantial effect on gene expression. We then evaluated the expression of cleaved Casp3, a marker for apoptosis and found a significantly increased number of Casp3-positive cells, as well as an enhanced Casp3 protein level in ethanol-exposed iPS cells (Fig. 1e and f). However, this ethanol-induced increase in apoptotic markers is not accompanied by any long-term changes in iPS cell proliferation rate (Fig. 1b). These data suggest that ethanol exposure in human iPS cells mediates an increase in inflammatory responses.

### Ethanol activates the inflammasome pathway in NPCs

NPCs are important for neurogenesis [22], and ethanol may affect their functions. In order to test this, we investigated the effects of ethanol on NPCs derived from iPS cells.

**Fig. 2:**
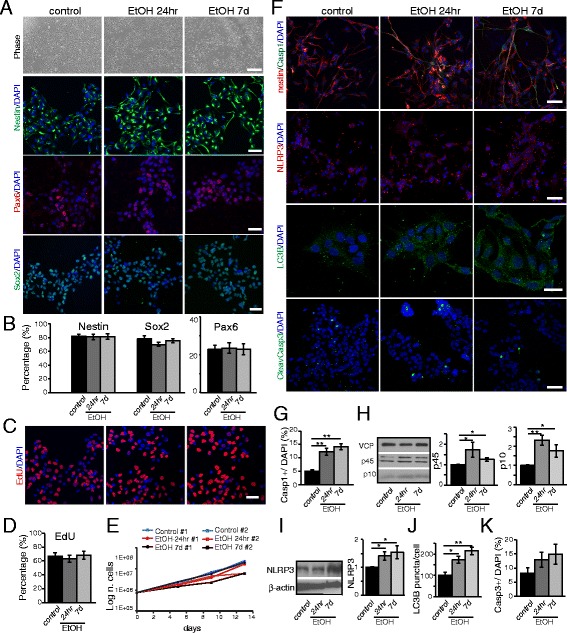
Ethanol activates the inflammasome pathway in NPCs. NPCs were obtained from iPS cells by epigenetic neural induction and treated with ethanol for 24hr or 7d. **a** From the top: phase contrast pictures show that the cell density between the different treatments is comparable at day 7. Scale bar: 200 μm. Immunofluorescence analysis by confocal microscopy shows the expression of neural multipotency markers Nestin, Pax6, and Sox2 in NPCs. Scale bar: 50 μm. **b** Graphs showing the relative percentage of Nestin^+^, Pax6^+^ and Sox2^+^ cells over the total number of DAPI^+^ nuclei. **c** Fluorescent images of EdU^+^ nuclei after incorporation assay for 24hr at day 7 of ethanol exposure. Scale bar: 50 μm. **d** Graph showing the relative percentage of EdU^+^ cells over the total number of DAPI^+^ nuclei. **e** Growth curve of NPC lines #1 and #2 after acute or chronic ethanol exposure. **f** Immunofluorescence analysis by confocal microscopy shows the expression of the inflammasome markers Casp1 and NLRP3, autophagy marker LC3B, and of the apoptotic marker Casp3 in NPCs with or without ethanol exposure (24hr or 7d). Scale bar: 20 & 50 μm (LC3B). **g** Graph showing the relative percentage of Casp1^+^ cells over the total number of DAPI^+^ nuclei. **h** Western Blot and graph showing the relative densitometric analysis of Casp1 (p45 and p10) expression. Quantification of relative protein expression normalized to VCP expression. **i** Western Blot and graph showing the relative densitometric analysis of NLRP3 expression. Protein quantification was normalized to β-actin. **j** Graph showing the relative percentage of LC3B puncta per cell. **k** Graph showing the relative percentage of Casp3^+^ cells over the total number of DAPI^+^ nuclei. The differences among all the values were not statistically significant unless indicated (* *p* ≤ 0.05, ** *p* ≤ 0.01, *** *p* ≤ 0.001). Student’s *t*-test was utilized for all experiments

Similar to what we found in the expression of pluripotent stem cell markers in iPS cells, we found no significant effects of ethanol on the expression of NPC markers, including Nestin, Pax6, and Sox2 (Fig. 2a and b). We then asked whether cell division is altered by alcohol exposure using 5-Ethynyl-2′-deoxyuridine (EdU) incorporation assays [23]. We found no statistically significant variation in the number of dividing cells in ethanol-exposed versus control cells (Fig. 2c and d). Likewise, ethanol had no effect on the number of self-renewing NPCs, as assessed by growth curve analysis (Fig. 2e).

The inflammasome pathway is known as an alternative to apoptosis, leading progressively to cell degeneration and death. Ethanol has been shown to activate this pathway in different cell systems [17, 24, 25], including mouse astrocytes [5], thus demonstrating the ability of neural cells to participate in the development of an inflammatory cascade. Given our data in iPS cells suggesting ethanol-induced cellular inflammation (Fig. 1), we sought to determine if ethanol activates the inflammasome pathway in NPCs. IHC analysis in NPCs revealed a robust increase of the number of Casp1^+^ cells after ethanol exposure, with no significant difference between 24hr vs. 7d exposure (Fig. 2f and g). Western Blot analysis revealed an increase in the expression of both the prodomain (p45) and the activated domain (p10) of Casp1 (Fig. 2h). This effect mirrored a similar increase in NLRP3 expression (Fig. 2f and i), thus confirming the involvement of the inflammasome pathway. Since the inflammasome-activated pathway is known to interplay with autophagy [6, 8, 9], we also evaluated the distribution of LC3B (a marker of the autophagosome membrane [26]) puncta in NPCs. Interestingly, the number of LC3B puncta was significantly increased in cells that were exposed to ethanol compared to control cells (Fig. 2f and j), suggesting that ethanol may also induce LC3B lipidation, an indication of involvement of the autophagy pathway. Finally, to determine whether ethanol could exert a pro-apoptotic effect on NPCs that could potentially impair neurogenesis [27], we determined the expression of Casp3. Though not significant, we detected an increased number of Casp3^+^ cells in ethanol exposed cultures compared to controls (Fig. 2f and k), suggesting that ethanol may condition long-term survival of progenitors and/or differentiating cells without an acute effect, and that pathways other than canonical apoptosis might be involved in AUD.

Taken together, these results support our hypothesis that activation of the inflammasome pathway is a major cellular event in both iPS cells and NPCs following exposure to ethanol.

### Pre-exposure to ethanol decreases the number of mature neurons derived from NPCs

**Fig. 3:**
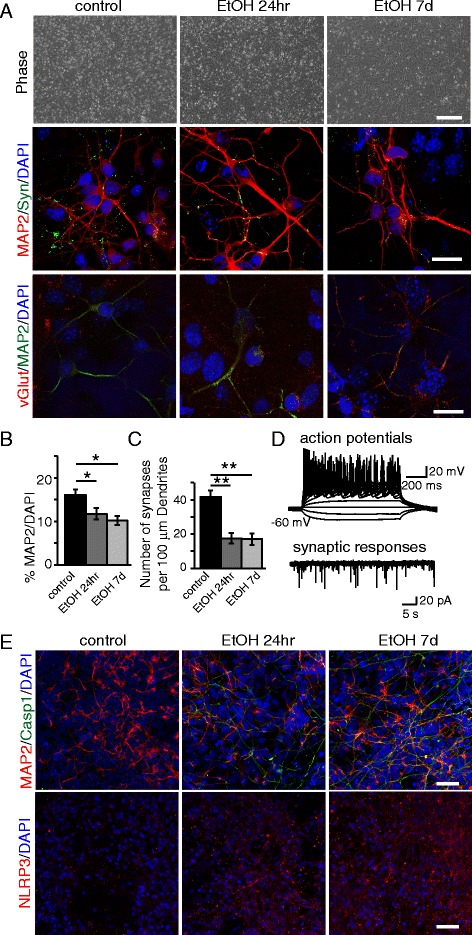
Early ethanol exposure leads to a decrease of NPC-derived neurons. NPCs were pretreated with ethanol for 24hr or 7d and differentiated to neurons for 26 days. **a** Phase contrast pictures of treated and untreated NPCs after 7 days of differentiation toward the neuronal lineage, and immunofluorescence analysis of neurons differentiated from NPCs showing the synaptic marker synapsin, and glutamatergic marker vGlut. Scale bars: 200 μm (*top*), 10 μm (*middle*, *bottom*). **b** Graph showing the relative percentage of MAP2^+^ cells over the total number of DAPI^+^ nuclei. **c** Number of synapses quantified by synapsin^+^ puncta per 100 μm dendrite length under control or ethanol pre-exposure conditions. **d** Electrophysiological analysis. **e** Immunofluorescence analysis showing the expression of Casp1, MAP2, and NLRP3. Scale bar: 50 μm. The differences among all the values were not statistically significant unless indicated (* *p* ≤ 0.05, ** *p* ≤ 0.01, *** *p* ≤ 0.001). Student’s *t*-test was utilized for all experiments

Since ethanol is teratogenic, and long-term effects may only become evident with time, we investigated whether pre-exposure to ethanol affects the differentiation of NPCs into neurons. Functional, mature neurons expressing MAP2, vGlut1, and synapsin were derived from the NPCs with or without ethanol pre-exposure (Fig. 3). Fewer mature neurons, identified by MAP2 expression, were generated from ethanol pre-exposed NPCs compared to untreated NPCs (Fig. 3a and b). Moreover, synaptic density appears to be reduced in neurons generated from ethanol treated NPCs when compared to neurons derived from control NPCs (Fig. 3a–c). However, in neurons derived from control and ethanol pre-exposed NPCs exhibited both repetitive action potentials as well as synaptic responses (Fig. 3d). Quite remarkably, it appears that inflammasome markers Casp1 and NLRP3 were prominent in neurons derived from ethanol pre-exposed NPCs (Fig. 3e). These findings suggest that neuronal differentiation is compromised (lower efficiency in maturation and less synapse formation) in NPCs pre-exposed to ethanol, and that neurons maintain certain cellular memory of neuroinflammation (i.e. with or without ethanol exposure) from the NPC stage.

### Ethanol alters mitochondrial and lysosomal patterns in both iPS cells and NPCs

**Fig. 4:**
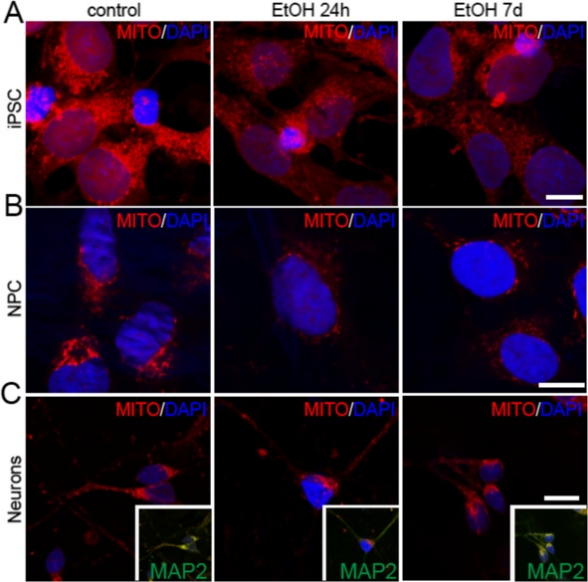
Ethanol alters mitochondrial patterns in iPS cells, NPCs, and NPC-derived neurons. Confocal microscopy images showing the mitochondrial pattern (Mitotracker) in iPS cells (**a**), NPCs (**b**), and NPC-derived neurons (after 26 days of differentiation) (**c**) after treatment with ethanol for 24hr or 7d. Nuclei (in blue) are counterstained with DAPI. *Inserts:* co-immunolabeling with MAP2 shows different colocalization of Mitotracker in neuronal cells. Scale bars: 10 μm

**Fig. 5:**
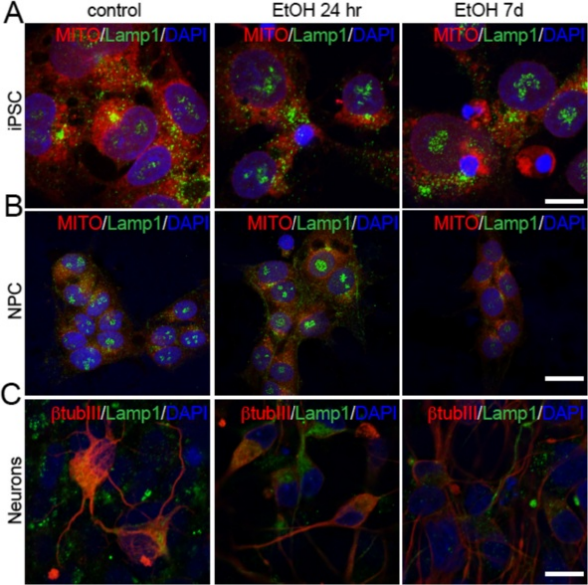
Ethanol alters lysosomal patterns in iPS cells, NPCs, and NPC-derived neurons. Analysis of co-localization of the lysosomal marker Lamp1 with Mitotracker in iPS cells (**a**) and NPCs (**b**) after 24hr or 7d treatment with ethanol, and co-localization of Lamp1 with β-tubulinIII in untreated and treated NPC-derived neurons (**c**). Scale bars: 10 μm (**a**), 20 μm (**b** and **c**)

Consistent with previous studies showing cross talk between the inflammasome pathway and lysosomal-mitochondrial machinery [8], we investigated the mitochondrial and lysosomal patterning in iPS cells and NPCs with or without ethanol exposure, as well as in neurons derived from NPCs with or without ethanol pre-exposure. In particular, we investigated the distribution of mitochondria and lysosomes using a Mitotracker Assay™ (Fig. 4) and IHC for the lysosomal marker Lamp1 (Fig. 5), respectively. Both mitochondria (Fig. 4) and lysosomes (Fig. 5) appear to be reduced in iPS cells or NPCs exposed to ethanol. In contrast to the more dispersed mitochondrial distribution in untreated iPS cells, NPCs, or neurons, the mitochondria cluster more prominently in the perinuclear area in treated cells, with a particularly robust effect in the 7d condition (Fig. 4a, b and 5a, b). Similar effects were observed in neurons derived from NPCs that were pre-exposed to ethanol (Fig. 4c and 5c).

### Ethanol pre-exposure increases the sensitivity of both iPS cells and NPCs to oxidative stress

**Fig. 6:**
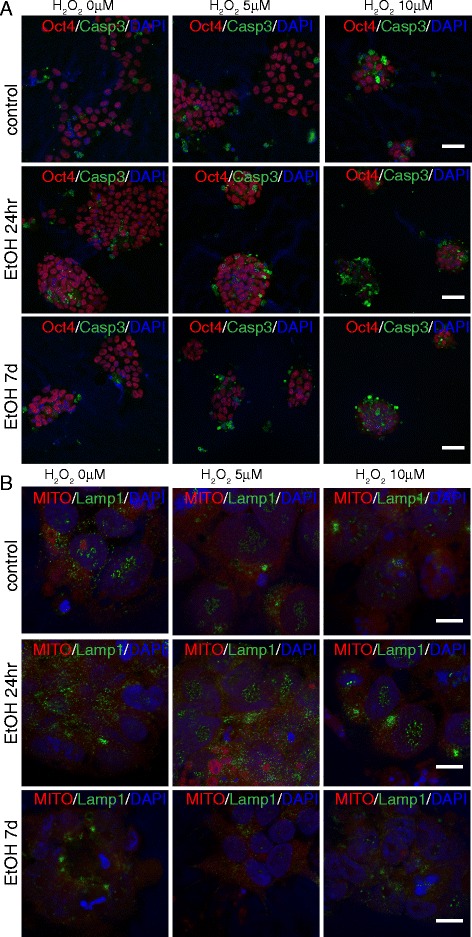
Cooperative effects of ethanol and peroxide challenges on apoptosis and lysosomal/mitochondrial distribution. At day 7 after exposure to ethanol for 24hr or 7d, iPS cells were exposed for 14hr to 5 or 10 μM H_2_O_2_ and immunostained with antibodies against Oct4 and Casp3 (**a**), or stained with Mitotracker^™^ and Lamp1 (**b**). Both single treatments with ethanol and H_2_O_2_ alter the normal patterns of the cell, but a remarkable enhancement of the effects observed following a single challenge is evident following a double challenge. Scale bars: 50 μm (**a**), 10 μm (**b**)

Previous studies have shown that alcohol abuse enhances neuroinflammation in vivo [15, 28, 29], with specific impairment of immune responses in an animal model of Human Immunodeficiency Virus-1 (HIV1) Encephalitis [28] and of neurological recovery after traumatic brain injury [29], thus suggesting that ethanol mediated-toxicity can exacerbate neuronal injury. Since damaging reactive oxygen species are generated during ethanol metabolism [30], and since the inflammasome pathway has recently been identified as player in a signaling response to a double challenge [24], we hypothesized that the ethanol-mediated activation of the inflammasome in iPS cells and NPCs would make them more vulnerable to a second toxic insult. To test this hypothesis in our system, we challenged both iPS cells and NPCs with peroxide (5 and 10 μM for iPS cells, and 100 and 500 μM for NPCs; concentrations were determined by the lethality of the exposure) for 14hr on day 7 after ethanol pre-exposure. The morphology of the cells that were challenged by peroxide was remarkably altered, becoming round and shrunken. This was accompanied by lysosomal and mitochondrial distributions that appeared clustered and inhomogeneous (Fig. 6b). Remarkably, this effect was enhanced in cells that had undergone both ethanol and peroxide treatments.

**Fig. 7:**
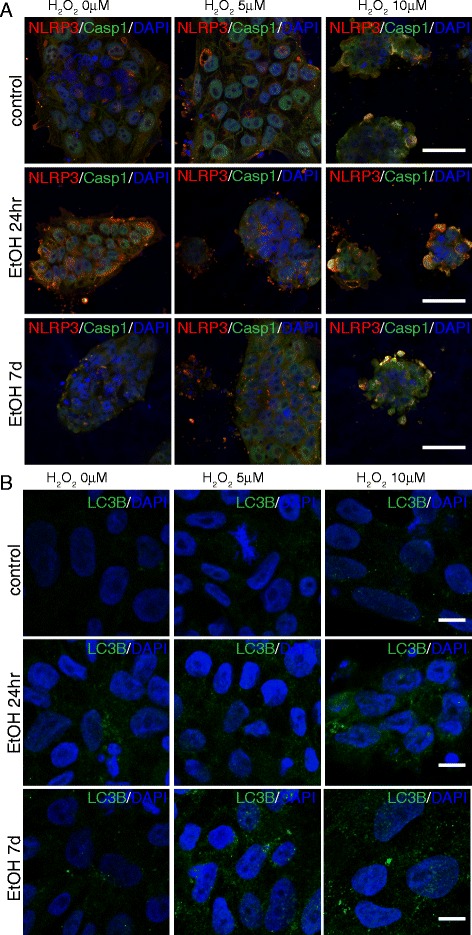
Cooperative effects of ethanol and peroxide challenges on inflammasome markers NLRP3 and Casp1 in iPS cells. On day 7 following 24hr or 7d ethanol treatment, iPS cells were exposed for 14hr to 5 or 10μM H_2_O_2_ and immunostained with antibodies against NLRP3 and Casp1 (**a**), and LC3B (**b**). At 10μM H_2_O_2_, iPS cells pretreated with ethanol for 7d displayed a dramatic increase in death. Scale bars: 50 μm (**a**), 10μm (**b**)

**Fig. 8:**
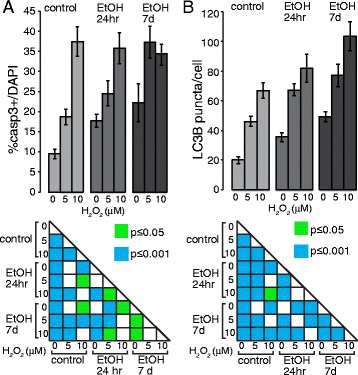
Cooperative effects of ethanol and peroxide challenges on Casp3^+^ cells and LC3B puncta in iPS cells. **a** Graph showing the relative percentages of Casp3^+^ cells over the total number of DAPI^+^ nuclei. *Low panel*s: Statistical significance was indicated. **b** Graph showing the relative percentages of LC3B puncta per cell. *Low panel*s: Statistical significance was indicated. Student’s *t*-test was utilized for all experiments

**Fig. 9:**
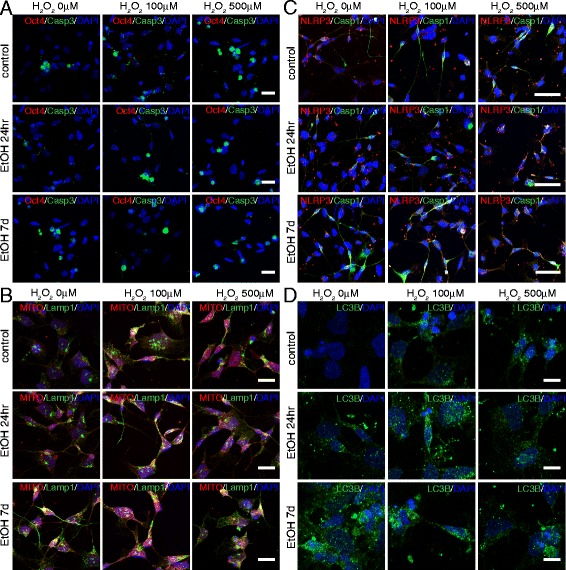
Cooperative effects of ethanol and peroxide challenges in NPCs. At day 7 after exposure to ethanol for 24hr or 7d, NPCs were exposed for 14hr to 100 or 500 μM H_2_O_2_ and immunostained with antibodies against Oct4 and Casp3 (**a**), NLRP3 and Casp1 (**c**), and LC3B (**d**), or stained with Mitotracker and Lamp1 (**b**). Both single treatments with ethanol and H_2_O_2_ alter the normal patterns of the cell, but a remarkable enhancement of the single effects is evident by the concurrence of the two treatments. Scale bars: 50 μm (**a**–**c**), 10 μm (**b**–**d**)

Accordingly, we observed a cumulative increase of the inflammasome-related markers Casp1 and NLRP3 (Fig. 7), of Casp3^+^ cells (Fig. 6a and 8a), and of LC3B puncta (Fig. 7b and 8b) in iPS cells that had undergone the double challenge compared to the single challenge (Fig. 6 and 7), showing that ethanol treatment induces long-term and long-lasting metabolic changes in the cell that can drive an enhanced response to any additional damage. On the contrary, while an increase in the number of Casp3^+^ cells was evident with peroxide or ethanol treatment alone in NPCs, no significant difference was detectable between cells that had undergone the double challenge compared to a single challenge (Fig. 9). This suggests that NPCs are more resilient than iPS cells to cumulative damages and/or that in our cell-based system the range of sensitivity is too narrow to reach statistical significance. Consistently, LC3B puncta appeared increased by each single challenge, but a quantitative evaluation in double challenged cells was impaired by the altered cell morphology. These data suggest that ethanol exposure in iPS cells and NPCs results in greater sensitivity to oxidative stress, which may contribute to the pathophysiology of neurogenesis in humans.

## Discussion

In this study, we show that exposure to ethanol at a physiological dose considered comparable to heavy alcohol usage in humans [31] activates the inflammasome pathway in human iPS cells and NPCs.

Although a wide array of studies has been performed on murine neural cells, controversial data have been obtained on the effects of ethanol on NSC proliferation or differentiation [3, 15, 32–34]. Whether a mouse model can mimic all aspects of human diseases is also highly controversial [35–37]. Nevertheless, in order to understand the pathophysiology of AUDs, it is imperative to elucidate the effects of ethanol on human cell function. Thus, we tested the impact of ethanol exposure on both iPS cells and NPCs derived from patients with no known history of alcoholism. Since it is virtually impossible to perfectly model the variable drinking habits and fluctuating blood alcohol concentration of an AUD patient [38], we opted to test acute (24hr) and prolonged (7d) alcohol exposure on iPS cells and NPCs. In particular, we chose to utilize an ethanol concentration of 70 mM, as this corresponds to the blood alcohol concentration of a heavy drinker, and treatments using concentrations above 100mM are known to cause a direct cytotoxic effect on the cells [38]. We chose a 24hr ethanol exposure to mimic binge alcohol drinking [34], and a 7d exposure (with daily replenishment of ethanol-containing medium) to recapitulate mild, recurring alcohol exposure. The gradual decrease in ethanol concentration in the medium due to evaporation in an unsealed culture dish follows a trend that is predicted to mimic the physiological oscillations of blood alcohol concentration in a heavy drinker with daily alcohol consumption [38–41]. Nevertheless, we acknowledge that it is possible that we didn’t achieve the intended effect of chronic, continuous alcohol exposure at higher doses. However, it is interesting to note that no difference was noticeable between acute and prolonged treatment in terms of markers for neuroinflammation, suggesting that an acute exposure to ethanol is sufficient to activate the inflammasome-mediated pathway.

Ethanol has no obvious effects on proliferation or morphology in either iPS cells or NPCs, but rather it primes the activation of pro-apoptotic pathways, such as those mediated by inflammasomes, as detected by increased staining for Casp3 and NLRP3. This is consistent with a previous study showing that chronic alcohol consumption does not affect cell proliferation in the human subventricular zone (SVZ) [15]. Given the strong interplay between the inflammasome and autophagosome pathways [8], and the effects of ethanol on the regulation of autophagy [9], we investigated the expression and distribution of LC3B, a protein incorporated into the autophagosomal membrane during their formation [42]. Interestingly, both iPS cells (Fig. 7b and 8b) and NPCs (Fig. 2f and j) displayed an increase of LC3B^+^ puncta (representative of autophagosomes and autophagosome-lysosome organelles). Accordingly, an anomalous distribution of Lamp1^+^ lysosomes was evident in treated cells. Thus, we suggest that an ethanol-mediated pro-apoptotic pathway additionally includes the participation of a disturbed autophagy process. Interestingly, it’s known that the inflammasome and autophagy pathways may participate independently of ethanol-mediated responses [43, 44]. However, given the multiplicity of pathways involved in both the regulation of autophagy and of the inflammasome, further studies will be necessary to dissect the different mechanisms engaged by ethanol-driven neuropathology. Along with the activation of the inflammasome pathway, an impaired elimination of defective mitochondria by selective autophagy (mitophagy) is among the most likely mediators of ethanol-induced damage [5, 9, 17]. Indeed, ethanol is known to cause damage to mitochondria resulting in the production of intracellular reactive oxygen species [4, 45, 46]. We found that mitochondrial distribution was altered in both iPS cells and NPCs, with a reduction in mitochondrial content, as well as an increased tendency to cluster in treated versus untreated cells. Nevertheless, no specific differences were detectable between the acute (24hr) or prolonged (7d) exposure [21, 32, 47]. Our findings suggest that even a very transient exposure to ethanol (24hr) will make both iPS cells and NPCs more sensitive to oxidative damage, resulting in a shift from a physiological homeostatic metabolism to an unbalanced state of inflammation, and possibly autophagy. The detailed cellular process involving autophagy, such as variations of Atg7 or Atg5, requires further studies.

Aside from being teratogenic, ethanol is known to exert its effects in a stage-specific manner [48]. If we assume that human iPS cells model early embryonic development, and NPCs represent both early and adult neurogenesis stages, we can speculate that exposure of the human brain to ethanol at a very early phase of gestation, likely due to maternal drinking habits, can lead to a pathological impairment of postnatal neurogenesis, such as causing FASD. On the contrary, exposure of an adult human brain to ethanol (i.e. under AUDs) generates variable grades of neuronal dysfunction, depending on age, genetic and epigenetic backgrounds. As a matter of fact, previous data reported a temporal susceptibility to ethanol neurotoxicity during development [49–51]. In accordance with this, we have shown that iPS cells are more sensitive to oxidative damage than iPS cell-derived NPCs, and that these effects are strongly exacerbated by a prior exposure to ethanol.

However, with the human adult brain, both age- and region-dependent effects of ethanol exposure have to be considered [51, 52]. A low grade pro-inflammatory phenotype has been shown to accompany aging in mammals, with a progressive disturbance of the interplay between autophagy and the inflammasome pathway [8]. Mitochondrial dysfunction and cortical spreading depression have been shown to follow ethanol abuse with aging [11]. Further studies should be performed in order to assess whether alcohol exposure in the adult human brain affects the mature or the neurogenic areas [15]. Additionally, the immunohistochemical analysis of the FASD human brain at the embryonic stage would provide additional information about ethanol-driven mechanisms from the pre-natal to the post-natal stage, including the participation of the inflammasome pathway in the early phase of FASD pathogenesis.

## Conclusions

Taken together, our observations emphasize that a disturbance in the cellular housekeeping, induced by alcohol exposure, can trigger the pro-inflammatory sensor NLRP3, therefore stimulating inflammatory reactions in pluripotent stem cells as well as NPCs. Evidence indicates that multiple factors play a role in the alcohol-mediated response, such as Heat Shock Factor 1 [53], mTOR [54, 55], or TLR4 [6]. Though a linear cascade of events has not yet been determined due to the concurrence of multiple cross-talking mechanisms, we have illustrated that an impairment of mitochondrial and lysosomal distribution and function are involved in ethanol-mediated induction of the inflammasome pathway, resulting in a disequilibrium of the innate immune response and metabolism. Moreover, we also conclude that ethanol differentially enhances the vulnerability of either pluripotent or neural progenitor cells to further challenge through the involvement of the NLRP3 inflammasome pathway that acts as a sensor of cell metabolism and that finally regulates cell survival and differentiation. A better understanding of neuronal metabolism shows promise in providing novel therapeutic avenues to tackle ethanol neurotoxicity. The iPS cell model represents an invaluable tool both for the identification of pathogenic mechanisms and for drug screening in human cells.

## Methods

### Generation of induced pluripotent stem cells and of neural progenitor cells

Three iPS cell lines were obtained from three healthy patients with no history of alcohol addiction. Briefly, cryopreserved primary lymphocytes were processed for CD4^+^ T-cell selection and Sendai viral reprogramming (CytoTune™, Life Tech), as described previously [56]. Pluripotency was confirmed by immunohistochemistry (IHC) for Oct4 and TRA-1-60 (data not shown). NPCs were generated from iPS cells by using the Gibco Neural Induction Medium (MAN0008031, Gibco) according to the manufacturer’s guidelines. iPS cells or NPCs after three passages from neural induction were treated with 70 mM ethanol for 24hr or 7d, by daily full replenishment with new culture medium. It has been reported that the alcohol concentration in culture gradually decreases, with an approximate 19-hr half-life in an unsealed culture dish [40]. The daily replenishment of the ethanol containing media, followed by a gradual loss by evaporation in unsealed culture dishes mimics the pattern of alcohol exposure of heavy drinkers [40]. After 7 days in vitro, the cells were plated at a density of 2.5×10^5^ on coverslips in 24-well plates for 24hr and processed for immunostaining, Western Blot, differentiation or peroxide treatment. All results are presented as relative to the average of the iPS cell lines respectively.

### Analysis of proliferation and generation of growth curves

The percentage of proliferating cells at 24hr was evaluated with an EdU [23] assay (Click-iT Plus EdU Imaging kit, MAN0009885, Molecular Probes, by Life Technologies) according to the manufacturer’s instructions. The rate of expansion of iPS cells and NPCs was determined by plating 7.5×10^5^ cells in separate wells of 6-well plates and by replenishing the media (mTeSR or expansion growth medium, respectively) daily. At each passage (p), the cells were harvested and the total cell number was calculated on the basis of amplification rate at each passage. The logarithmic value of the total viable-cell number was plotted against the days, starting from the beginning of the experiment. For each condition, growth curves were performed in duplicate, but a representative curve is reported.

### Electrophysiology

Standard electrophysiology was performed as described by Vierbuchen et al. [57] and Pang et al. [58]. Spontaneous post-synaptic currents (PSCs) were obtained from neurons with the resting membrane potential held at −70 mV. Both current and voltage clamp experiments were performed. For step current experiments, membrane current was held at 0 pA. 5 pA current injections were provided from −20 pA to 35 pA. In voltage clamp experiments, resting membrane voltage was held to -60mV. Step voltage injections were given from −100 mV to 0 mV with a step size of 10 mV. Action potentials and synaptic currents were monitored with Multiclamp 700B amplifier (Molecular Devices). Clampex 10 data acquisition software (Molecular devices) was used for collecting data. The whole-cell pipette solution contains (in mM) 126 K-Gluconate, 4 KCl, 10 HEPES, 0.3 Na_2_-GTP, 4 Mg-ATP and 10 Phosphocreatine (pH 7.2, adjusted with KOH). The bath solution contains (in mM) 140 NaCl, 5 KCl, 2 MgCl_2_, 2 CaCl_2_, 10 HEPES, 10 glucose (pH 7.4, adjusted with NaOH).

### Mitochondrial pattern analysis

The analysis of mitochondrial distribution was performed by staining fixed cells with 300nM Mitotracker Red CMXRos (Life Technologies) for 20 min, according to the manufacturer’s protocol. Micrographs were taken with a Zeiss LSM700 confocal microscope.

### Neuronal differentiation

NPCs were plated as described above. After 24hr in expansion medium, NPCs were cultured for 26 div in Neurobasal medium containing B27, BDNF (10nM) and NT3 (10nM). 2/3 of the total volume of medium was replenished every 5–6 days. In particular, after 10 days from plating, differentiating NPCs were plated over mouse glia at a density of 50000/cm^2^ and cultured for an additional 3 weeks.

### Immunocytochemistry

Cultures were fixed in freshly prepared, buffered 4% paraformaldehyde. After blocking with 20% normal goat serum and permeabilization for 10min with 0.2% Triton X-100 in PBS, cultures were incubated overnight at 4°C with the following antibodies (mAb, monoclonal; pAb, polyclonal): Nestin ( MAB5326, Millipore, 1:200), Ki67 (ab16667, Abcam, 1:1000), Oct4 (MAB4401, Millipore, 1:2000), Pax6 (Developmental Studies Hybridoma Bank; 1:30), β-tubulin isotype III (β-tubIII, mAb, MMS-435P Covance, 1:400), Glial Fibrillary Acidic Protein (GFAP, z033429-2, Dako, 1:400), Lamp1 (pAb, ab24170, Abcam, 1:750), Cleaved Caspase-3 (pAb, #9661, Cell Signaling, 1:500), NLRP3 (pAb, AG-20B-0014-C100, AdipoGen, 1:200), Microtubule-Associated Protein 2 (MAP2, AB5622, Millipore, 1:400), Caspase 1 (Casp1, SC-515, Santa Cruz Biotechnology, 1:100), LC3B (pAb#2775, Cell Signaling, 1:400), GAPDH (ab9485, Abcam, 1:3000). After removal of the primary antibodies and repeated washes with PBS, cultures were incubated at room temperature for 45 min with secondary antibodies labeled with Alexa Fluor 633 or 488 (anti-mouse and/or anti-rabbit, Molecular Probes). Samples were then labeled with DAPI (0.3 μg/ml, Roche) for nuclear staining and rinsed with PBS for mounting and analysis. Microphotographs were taken using a confocal microscope.

### Western blot analysis

Immunoblots were performed as described [59]. Membranes were incubated with rabbit antibodies against Lamp1, Caspase 1, Caspase 3, and NLRP3 (see immunocytochemistry analysis for dilution). Bands were quantified by densitometric analysis of the ECL-exposed films. The samples derived from the same experiment and gels/blots were processed in parallel.

### Statistical analysis

Growth curve analysis was performed on two independent cell lines. All other experiments were performed at least three independent times by using three different cell lines. Data are presented as bar graphs. Statistical analysis was performed by *two sample independent t* test. Data are reported as mean ± standard error of mean (SEM). *P < 0.05. **P < 0.01, ***P < 0.001.

### Ethics approval and consent to participate

All animal procedures were approved by Rutgers University Robert Wood Johnson Medical School Institutional Animal Care and Use Committee (IACUC). No human subjects were included in this study.

### Consent for publication

Not applicable.

## Abbreviations

AUD: alcohol use disorder
BDNF: brain derived neurotrophic factor
Casp: caspase
CD4: T cell surface glycoprotein
CDC: centers for disease control and prevention
CNS: central nervous system
DAPI: 4′-6-diamidin-2-phenylindole
EdU: 5-Ethyniyl-2′-deoxyuridine
ESC: embryonic stem cells
FASD: fetal alcohol spectrum disorder
GAPDH: glyceraldehyde-3-phosphate Dehydrogenase
GDNF: glial derived neurotrophic factor
GFAP: glial fibrillary acidic protein
HIV1: human immunodeficiency virus-1
Iba1: ionized calcium-binding adapter molecule 1
IHC: immunohistochemistry
iPS: induced pluripotent stem
Lamp1: lysosomal associated membrane protein 1
LC3B: microtubule associated protein 1A/1B-light chain 3
MAP2: microtubule associated protein type 2
mTOR: mammalian target of Rapamycin
NIH: National Institutes of Health
NLRP3: nucleotide-binding oligomerization domain-like receptor family pyrin domain containing 3
NPCs: neural progenitor cells
NT3: neurotrophin 3
Oct4: octamer binding transcription factor 4
Pax6: paired box protein 6
PBS: phosphate buffered saline
Sox2: sex determining region Y-box 2
SVZ: sub ventricular zone
TLR: toll-like receptor
Tra-1-60: T cell receptor alpha locus-1-60
β − tub III: class III beta-tubulin
